# Diffuse xanthomas in a patient with lipoprotein X hyperlipidemia

**DOI:** 10.1016/j.jdcr.2023.07.021

**Published:** 2023-07-27

**Authors:** Katherine Grace Byrnes, Scott Berg, Lydia Luu, Lisa Borretta, Richard Hal Flowers

**Affiliations:** a University of Virginia School of Medicine, Charlottesville, Virginia; b Department of Dermatology, University of Virginia, Charlottesville, Virginia; c Department of Pathology, University of Virginia, Charlottesville, Virginia

**Keywords:** diffuse xanthomas, lipoprotein X hyperlipidemia, xanthelasma, xanthomas

## Introduction

Cutaneous xanthomas are characterized by benign lipid deposits in the dermis. Clinical variants include eruptive xanthomas, tendinous xanthomas, tuberous xanthomas, plane xanthomas, and verruciform xanthomas. Xanthomas can be idiopathic but typically occur in association with inherited or acquired dyslipidemias. Although cutaneous xanthomas, particularly xanthelasma, are common lesions seen by dermatologists, patients rarely present with diffuse xanthomas.

Lipoprotein X (LpX) is an abnormal structurally distinct lipoprotein indicative of cholestasis and is seen in liver diseases, such as primary sclerosing cholangitis (PSC).^1^ LpX formation is a nonfamilial cause of hyperlipidemia, and while the pathophysiology and epidemiology of LpX-induced hyperlipidemia have not been fully elucidated, therapy relies on treatment of the underlying cholestatic disease.^1^ Here, we report a case of diffuse xanthomas presenting in a patient with LpX hyperlipidemia.

## Case report

A 30-year-old woman with PSC, Crohn’s disease, and LpX hyperlipidemia presented for evaluation of new-onset pruritic skin lesions on shoulders and arms. She reported that her rash started 3 weeks before her presentation in the clinic. She had attempted to treat the lesions with topical steroids without improvement. She had a history of biopsy-confirmed reactive perforating collagenosis and granuloma annulare on the trunk and extremities for which she was previously followed.

**Fig 1:**
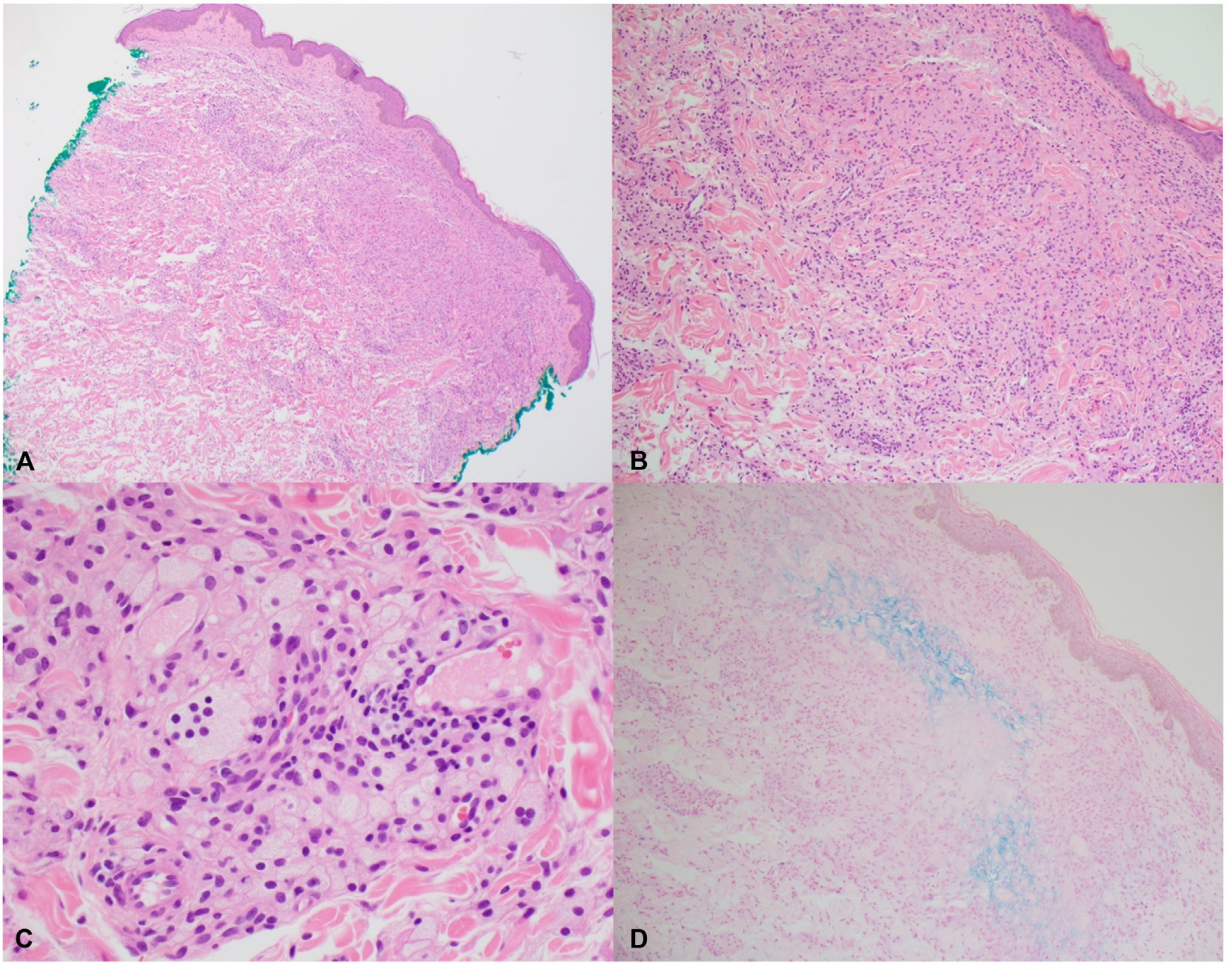
Biopsy from the right side of the neck. Hematoxylin and eosin stain (**A**, **B**, and **C**) showing brisk proliferation of xanthomatized histiocytes and reactive granulomas in the dermis and Alcian Blue stain **(D)** highlighting increased mucin deposition. (**A**, **B**, and **C**, Hematoxylin-eosin stain; original magnifications: **A,** 4×; **B,** 10×; **C,** 40×; **D,** Alcian Blue stain; original magnification: 10×.)

On examination, yellowish to hypopigmented coalescing papules and small plaques were present on the bilateral neck extending onto the shoulders and upper portion of the arms. She also had diffuse follicular hyperpigmented papules over the bilateral lower portion of the legs, bilateral upper portion of the legs, back, and bilateral arms, which were consistent with her prior reactive perforating collagenosis diagnosis. A punch biopsy from the skin of the patient’s right side of the neck showed a brisk proliferation of xanthomatized histiocytes in the papillary, mid, and reticular dermis, with foci of necrobiosis and mucin deposition (Fig 1). Mucin deposition was attributed to concomitant granuloma annulare. Extracellular deposition of lipid was present.

Given these clinical and histologic findings, cutaneous xanthomas were diagnosed. The lipid panel at the time revealed a total cholesterol level of 876 mg/dL (reference range: <200 mg/dL), a low-density lipoprotein level of 840 mg/dL (reference range: <130 mg/dL), and triglycerides within normal limits. The patient’s PSC was active around this time and was managed by hepatology. She underwent endoscopic retrograde cholangiopancreatography a month later for biliary stricture, and a biliary sphincterotomy was performed. The procedure was complicated by a biliary leak, and over the next few months, the patient began pretransplant screening given her progressive liver disease.

**Fig 2:**
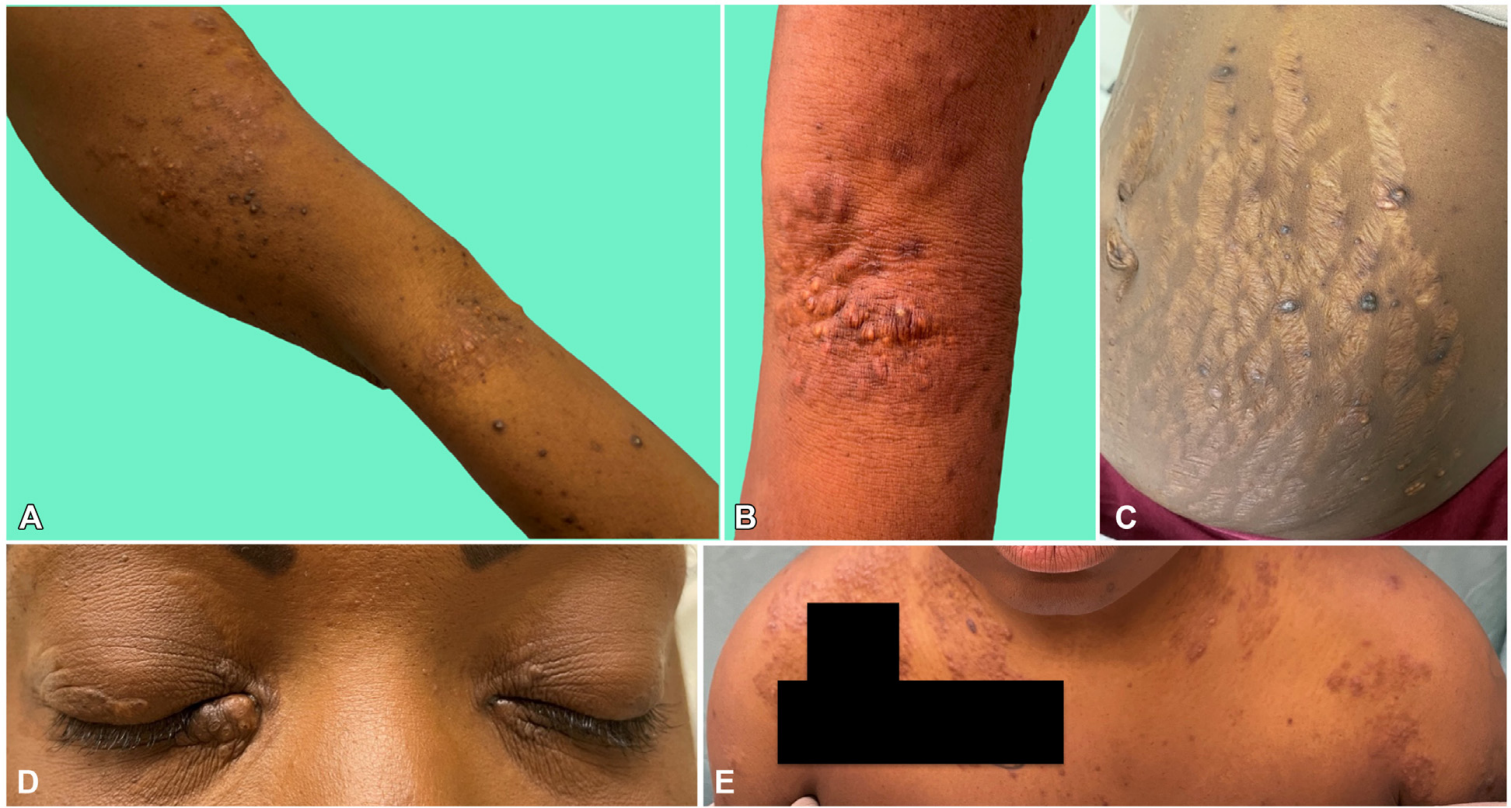
Cutaneous xanthomas on the patient’s right upper extremity **(A)**, left antecubital fossa **(B)**, abdomen **(C)**, eyelids **(D)**, and chest **(E)**.

On follow-up 6 months after presentation with dermatology, the xanthomas had progressed extensively to involve the eyelids, upper portion of the chest, upper extremities, including antecubital fossae, and abdomen, including abdominal striae. She had scleral icterus, pink-brown with subtle yellow papules and nodules on the bilateral upper and lower eyelids, with the largest nodule on the medial aspect of the right lower eyelid, and pink-brown with subtle yellow papules and nodules on the upper portion of the chest, anterior aspect of the shoulders, antecubital fossae, and extensor elbow surfaces of the bilateral upper extremities and bilateral lower portion of the abdomen (Fig 2).

At this time, our patient’s total cholesterol and low-density lipoprotein had increased to 2011 mg/dL and 1938 mg/dL, respectively, despite treatment with the maximal tolerated oral dose of cholesterol-lowering medication (12 g of cholestyramine daily). At this time, she began plasmapheresis every 2 weeks, which markedly decreased her total cholesterol level to 196 mg/dL and low-density lipoprotein level to 178 mg/dL over 2 months of treatment. She recently received a liver transplant for her PSC, which resolved her LpX-mediated hyperlipidemia and markedly improved her cutaneous xanthomas.

## Discussion

We report a rare case of diffuse xanthomas presenting in a patient with LpX-mediated hyperlipidemia. LpX as a cause of cutaneous xanthomas has been rarely reported.^2^, ^3^, ^4^, ^5^, ^6^ Both LpX-induced hypercholesteremia^7^ and isolated LpX^2^ increase lipid accumulation in human monocyte and human monocyte-derived macrophage experiments, respectively. LpX hyperlipidemia, secondary to PSC, was likely the cause of our patient’s diffuse xanthomas.

Limited descriptions of cutaneous xanthomas in the setting of LpX exist in the literature. Generally, the xanthomas reported have had similar morphologies to those of our patient but have been distributed less diffusely. We have summarized these cases in Table I.^3^^,^^4^^,^^7^, ^8^, ^9^ Three of the 6 reported patients with cutaneous xanthomas and LpX hyperlipidemia had primary biliary cholangitis, whereas 2 had PSC. All 6 reported cases were women, with ages ranging from 28 to 51 years. In the only other well-documented case of diffuse xanthomas secondary to LpX we have identified, the patient experienced resolution of pruritus and notable improvement of xanthomas 5 months after liver transplant.^8^ Of the 3 patients treated with plasmapheresis to manage their LpX-related lipid abnormalities, all 3 experienced improvement or complete regression of their xanthomas.^3^^,^^7^^,^^9^

**Table I:** Comparison of reported cases of xanthomas in patients with lipoprotein X hyperlipidemia

Case	Age (y)	Sex	Underlying disease	Distribution and morphology of cutaneous disease	Outcome
Brandt et al, 2015^3^	51	Female	PSC	Auricles, nostrils, palmar creases: small white-yellowish (“planar xanthomas”)	Resolution 4 wks after completion of plasmapheresis
Kattah et al, 2019^4^	28	Female	PBC	Perioral: multiple small yellowish papules with smooth, shiny surface and regular, well-defined borders Interdigital, dorsal hand: white-yellowish, confluent, smooth-surface papules with irregular borders Eyelids: xanthelasma	Slow improvement with reduction in number and severity of papules from pharmacologic management of dyslipidemia
Lian et al, 2020^7^	36	Female	PBC and autoimmune hepatitis	Right upper extremity: white-yellowish smooth eruptive and planar xanthomas	Reduction in lesions after 20 wks of biweekly plasmapheresis treatments
Dang et al, 2021^9^	27	Female	cholestatic fulminant hepatitis	Palmar creases: discrete yellowish papules (“xanthoma striatum palmare”) Abdomen, back, limbs, face: 1-2 cm scaly mildly erythematous papules and plaques (“verrucous plane xanthomas”)	Resolution after 18 wks of plasmapheresis treatments
Harris et al, 2021^8^	33	Female	PBC	Intertriginous areas, extensor joints of the hands and feet, palmar creases, trunk: innumerable soft yellow-brown papules distributed symmetrically, coalescing into plaques Dorsal aspect of the fingers: multiple soft, yellow-brown papules and nodules (“tuberous xanthomas”) Eyelids: soft yellow-brown plaques in the periorbital areas (“xanthelasma”)	Improvement of xanthomas 5 mo after liver transplant
Present case	30	Female	PSC	Eyelids: pink-brown with subtle yellow papules and nodules Upper portion of the chest, anterior shoulders, antecubital fossae, extensor elbows, lower portion of the abdomen (including abdominal striae): pink-brown with subtle yellow papules and nodules	Improvement of xanthomas after liver transplant

*PSC*, Primary Sclerosing Cholangitis; *PBC*, primary biliary cholangitis.

Although cases of xanthomas secondary to LpX hyperlipidemia are rare in general, we identified only one other case reporting such diffuse distribution.^8^ Moreover, the present case is highly distinct in the involvement of abdominal striae.

In conclusion, we report a very rare case of diffuse cutaneous xanthomas in a patient with LpX hyperlipidemia. Clinicians should be aware of this rare etiology of cutaneous xanthomas and know that often apheresis and liver transplant are necessary to correct the underlying hepatobiliary derangement.

## Conflicts of interest

Dr Flowers receives clinical trial funding from Concert Pharmaceuticals, 10.13039/100006483AbbVie, and 10.13039/100009857Regeneron Pharmaceuticals. Drs Berg, Luu, Borretta and author Byrnes have no conflicts of interest to declare.
